# Flexible adjustment of anticipations in human outcome processing

**DOI:** 10.1038/s41598-022-12741-0

**Published:** 2022-05-27

**Authors:** Selim Habiby Alaoui, Alexandra Adam-Darqué, Armin Schnider

**Affiliations:** Laboratory of Cognitive Neurorehabilitation, Division of Neurorehabilitation, Department of Clinical Neurosciences, University Hospital and University of Geneva, Av. de Beau-Séjour 26, 1205 Geneva, Switzerland

**Keywords:** Decision, Cognitive neuroscience, Reward, Neural decoding, Learning and memory, Cortex, Extinction, Neuroscience, Human behaviour

## Abstract

To sense whether thoughts refer to current reality or not, a capacity called orbitofrontal reality filtering, depends on an orbitofrontal signal when anticipated outcomes fail to occur. Here, we explored the flexibility and precision of outcome processing in a deterministic reversal learning task. Healthy subjects decided which one of two colored squares hid a target stimulus. Brain activity was measured with high-density electroencephalography. Stimuli resembling, but not identical with, the target stimuli were initially processed like different stimuli from 210 to 250 ms, irrespective of behavioral relevance. From 250 ms on, they were processed according to behavioral relevance: If they required a subsequent switch, they were processed like different stimuli; if they had been declared potential targets, they were treated like true targets. Stimuli requiring a behavioral switch induced strong theta activity in orbitofrontal, ventromedial, and medial temporal regions. The study indicates flexible adaptation of anticipations but precise processing of outcomes, mainly determined by behavioral relevance.

## Introduction

Recent years have seen a surge in studies on the role of the orbitofrontal cortex (OFC) in behavioral control. Its abilities to represent the content of expected outcomes (e.g., type of expected reward), to calculate and adapt their current value^1–7^, to signal their occurrence or absence^8,9^, and to enable behavioral switches^10^ make it one of the main cortical centers for behavioral adaptation and decision making^11–13^.

A distinct OFC function that has gone relatively unnoticed concerns the capacity to synchronize thought and behavior with ongoing reality. Patients who fail in this capacity confuse where they are and what their current role is, act on the basis of ideas that have no relation with current reality (they typically enact previous habits) and justify their presently inappropriate acts with confabulations^14–16^. Their lesions involve the posterior medial OFC (area 13), or structures directly connected with it^17,18^. Two experimental tasks have separated these patients from amnesic subjects that did not confuse reality: (1) when performing repeated continuous recognition tasks, they increased their false positive rate from run to run; they failed to notice that familiarity with items did not refer to the currently ongoing run, that is, current reality^14–16^; (2) in a deterministic reversal learning task, they abnormally often continued to choose the cue that was no longer followed by the expected outcome; that is, they failed to normally integrate the absence of the expected outcome into their thinking^16,19^. Healthy subjects correctly performing these tasks activated the posterior medial OFC (area 13)^20–23^. Correct processing of the stimuli on which the reality confusing patients failed induced a frontal positivity at about 200–300 ms in both tasks^24–26^.

In a reversal learning task, this frontal potential only appeared when the absence of the expected outcome was behaviorally relevant, that is, when it indicated a need to switch to the alternate cue on the next trial. It neither occurred upon presentation of an unexpected (behaviorally irrelevant) stimulus^25^, nor in response to (behaviorally irrelevant) absence of reward^27^, nor when it had ambiguous behavioral relevance in a probabilistic task^28^. Such situations may, however evoke other potentials associated with error processing and behavioral switches. The feedback-related negativity (FRN), a negative wave peaking at about 300 ms over central electrodes, has been described in response to conflicting outcomes differing from expectation^29–33^. Unexpected outcomes may further evoke a P300, a positivity over central electrodes peaking around 400–500 ms, which seems to predict behavioral adaptation^31,34^ and to reflect context updating^31,35,36^.

The hypothesis then is that the human (probably also non-human) OFC produces an early, brief (200–300 ms) signal—akin to an extinction signal—whenever an upcoming thought finds no correlate in ongoing reality and thus represents a pure thought or fantasy. We call this mechanism Orbitofrontal Reality Filtering (ORFi)^18,37^.

How precise is this mechanism? What does the brain—or OFC—accept as a thought that is consistent with reality? The studies mentioned above, indicating that the OFC represents precise outcomes but adapts their value to the present context^1,2,5^, suggest both precise and flexible outcome monitoring. Here, we tested the hypothesis that the human brain flexibly adapts the breadth of anticipations but then very precisely evaluates their occurrence or absence. Specifically, we expected that the absence of distinct stimuli would evoke, or not, a frontal positivity at 200–300 ms solely depending on their declared behavioral relevance rather than their physical resemblance to target stimuli.

## Results

### Behavioral results

**Figure 1:**
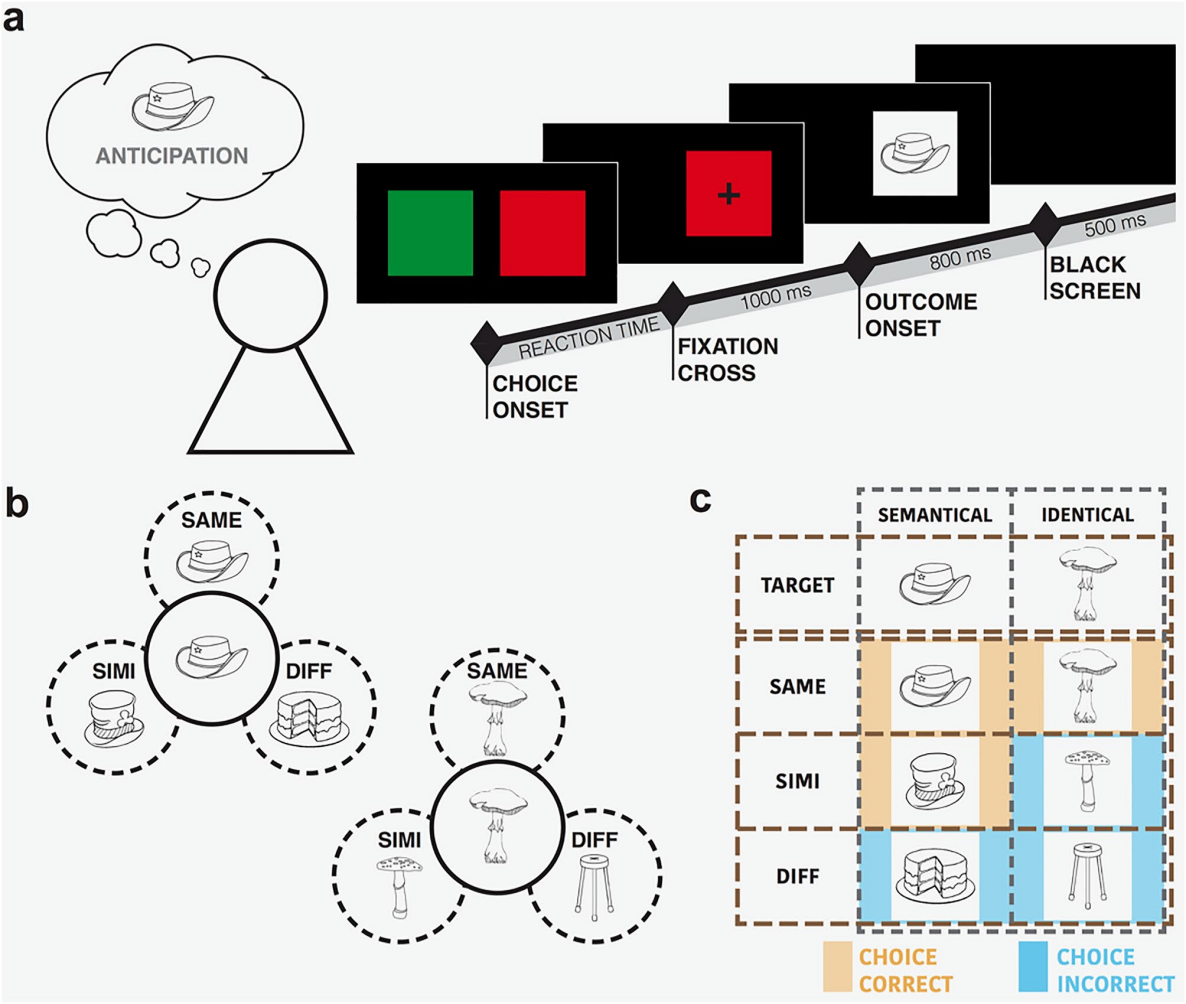
Task’s design. (**a**) Time line of the trial. It starts with the presentation of two colored squares (choice onset). A fixation cross appears as soon as the subject has selected a square, followed by the presentation of the outcome (outcome onset). The trial ends with a black screen. (**b**) Example of three different categories of possible outcomes. SAME represents the identical object, SIMI represents a semantically similar, but not identical object, and DIFF represents an object from another semantic group. (**c**) Categorization of the outcomes, according to the condition, the type of object and the required subsequent behavior: orange indicates stimuli after which subjects have to continue to choose the same square while a blue indicates stimuli that should induce a switch on the next trial.

In the present study, we used high-density evoked potentials in 22 healthy human subjects during a deterministic reversal learning task, similar to the one used in previous clinical and imaging studies^16,19,21,25^, to explore the precision of the outcome monitoring mechanism presumably underlying ORFi (Fig. 1). Subjects had to decide which one of two colored squares hid the design of an object and to continue to choose the same square (cue) as long as the anticipation of the target design truly appearing was confirmed by the outcome (Fig. 1a). There were three possible outcomes: (1) appearance of the target object itself, designated SAME, (2) an object from the same semantic group and closely resembling the principal target object, designated SIMI (for “similar”), or (3) an unequivocally different object, bearing no resemblance to the target object, designated DIFF (Fig. 1b,c). Subjects were instructed to switch to the alternate square on the next trial when the target object failed to appear, as indicated by the appearance of another object.

There were two experimental conditions. In one condition—termed “identical”—the target object was individually designated (“choose the square that hides THIS hat.”); appearance of a related object (a similar hat) would correspond to the absence of the expected outcome and should induce a switch on the next trial. In the other condition—termed “semantical”—the target object was defined as a semantic group (“choose the square that hides A hat.”). In both conditions, the principal target object was repeated six times more often (as SAME) than both the SIMI and DIFF stimuli. This was done to strongly bias the anticipation towards the primary target stimulus (SAME).

We expected that the electrophysiological reaction to appearance of the similar, but not identical, object would differ flexibly according to the precise task condition. Thus, the appearance of a similar object would induce the positive frontal potential at 200–300 ms and additional electrophysiological elements typical of ORFi only if it represented the absence of the target object defined for the task condition, thus signalling a need to switch to the alternate square on the next trial.

Subjects performed successfully in both conditions, with a correct choice in 98 ± 1.2% of trials in the “semantical” condition and 97.2 ± 1.3% in the “identical” condition. Conversely, they incorrectly selected the alternate square after a correct choice (association error) in 1 ± 0.7% of trials in the “semantical” and 1.4 ± 0.6% in the “identical” condition. In addition, they continued to choose the incorrect square (extinction error) in 8.1 ± 5.9% of trials in the “semantical” and 6.4 ± 3.7% in the “identical” condition. There was a minor influence of the outcomes on the reaction time, with a significant main effect of trials (*F*_(1.49,31.31)_ = 5.86, *p* = 0.012, *η*^2^ = 0.22). In the “identical” condition, participants were slightly faster following a SIMI (469 ± 148 ms) than SAME (482 ± 149 ms, *t*_(21)_ = 3.06, *p* < 0.05, Cohen’s *d* = 0.09) or DIFF outcome (506 ± 162 ms, *t*_(21)_ = 2.88, *p* < 0.05, Cohen’s *d* = 0.24). No significant difference was found in the condition “semantical” (SAME, 460 ± 133 ms; SIMI, 466 ± 154 ms; DIFF, 478 ± 161 ms).

### Distinct electrophysiological potential when outcomes indicate need to switch behavior

**Figure 2:**
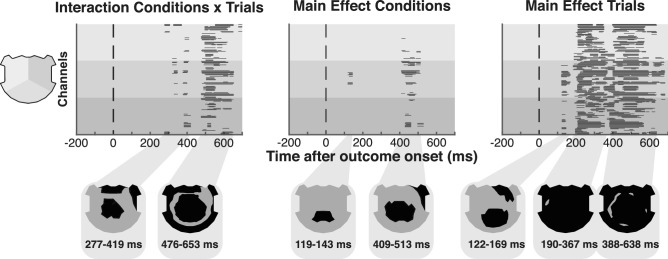
Global waveform analysis. Results of time-wise amplitude differences over all 156 electrodes show the interaction between condition and trials (left), the main effect of condition (middle) and the main effect of trials (right) of the 2 × 3 rmANOVA. Electrodes with significant differences (p < 0.01; ≥ 20 ms) are displayed in black.

Brain activity was recorded using high-density electroencephalography. We first evaluated whether unexpected outcomes (appearance of another than the target object) induced distinct event-related potentials (ERPs) in the “semantical” and “identical” conditions over the whole set of electrodes. We observed different time periods of significant ERP amplitude differences at an early and late stage of outcome processing. A time and electrodes-wise 2 × 3 repeated measure ANOVA with the factors condition (semantical vs. identical) X trial type (SAME, SIMI, DIFF) performed over all electrodes revealed a significant interaction at 277–419 ms and at 476–653 ms after the outcome onset (Fig. 2, left). There was a brief main effect of condition at 409–553 ms (Fig. 2, middle). Main effects of trial type were present at 120–169 ms, then extended from 190 to 367 ms and from 388 to 638 ms (Fig. 2, right).

These findings are consistent with previous studies which demonstrated changes in potential amplitudes over the frontal regions at an early stage (around 200–300 ms) and over the central and posterior regions at a late stage (around 400–600 ms) of processing following the absence of the expected outcome^25,27,38^.

Previous studies had shown main effects of outcome processing over frontal, central end posterior electrodes^25,27,28,39^. Therefore, we next explored ERP waveform at three clusters of five electrodes each over these regions.

**Figure 3:**
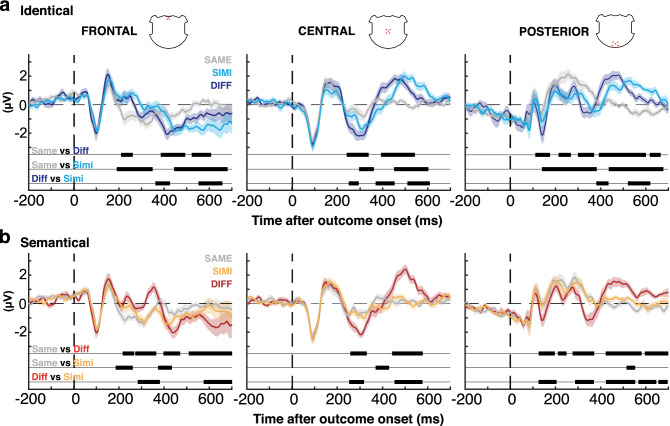
Cluster waveforms analysis. The curves show averaged potentials evoked by presentation of outcomes SAME, SIMI and DIFF, in condition identical (**a**) and condition semantical (**b**), at three clusters of five electrodes; frontal (left), central (middle) and posterior (right).

In the condition “identical” (“choose THIS hat”), SIMI and DIFF stimuli—both of which require a subsequent switch—induced largely similar responses, which differed from SAME. In particular, SIMI and DIFF induced a frontal positivity in comparison to the negative deflection induced by SAME at about 200–300 ms (Fig. 3a, “Identical”), plus an extended positivity over the central and posterior clusters at about 400–600 ms.

In the condition “semantical” (“choose A hat”), DIFF stimuli—the only ones requiring a switch—induced a frontal positivity different from SAME stimuli from 214 to 364 ms (Fig. 3b). SIMI stimuli initially sided with the DIFF stimuli (184–260 ms) to immediately join the potential induced by SAME and thus differing from DIFF between 283 and 380 ms (p < 0.05; cluster corrected; Fig. 3b). Over the central and posterior electrode clusters, SIMI induced virtually similar amplitudes as SAME. Over these electrodes the main difference was between a strongly positive response to DIFF at about 400–600 ms in comparison to SAME and SIMI. Thus, SIMI stimuli very rapidly, and then consistently, induced the same potential as SAME, consistent with their current significance as acceptable outcomes, thus requiring no switch of behaviour.

Thus, in both conditions, ERP differences were concordant with the behavioural relevance of the outcomes. Stimuli that required a switch in the subsequent trial evoked a frontal positivity at about 190–360 ms, compatible with the ORFi potential observed in earlier reality filtering^24,26^ and outcome monitoring tasks^25,27,38^, however, with a twist: in the semantical condition, SIMI were initially (184–260 ms) treated as different (similar to DIFF), but then immediately joined the potential evoked by SAME, which has similar behavioural significance, i.e., to stay with the previously chosen rectangle. The consequence was a slight delay of the early frontal positivity in response to SIMI (283–380 ms; Fig. 3b, semantical, frontal). This suggests a rapid process detecting dissimilarity between the presented and the expected stimulus irrespective of its behavioral significance^40^.

In both conditions, this frontal positivity at 190–360 ms partly overlapped towards its end with a central negativity in response to outcomes, which required a switch (Fig. 3a,b, frontal, central).

### Difference between “similar” outcomes with distinct behavior meaning

**Figure 4:**
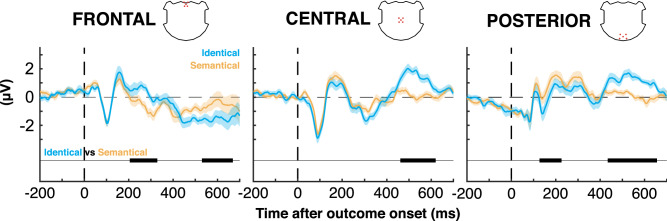
Cluster waveform comparison between outcomes SIMI of identical (bleu) and semantical (orange) conditions, at the three clusters: frontal (left), central (middle), posterior (right).

Of special interest to this study is the direct comparison of the electrophysiological responses to SIMI stimuli across the two conditions; only in the condition “identical” do these stimuli require a switch on the next trial. Figure 4 describes this comparison. Evoked potentials differed at both early and late stages of processing (Fig. 4). In condition “identical”, SIMI stimuli evoked a significantly stronger positivity than in condition “semantical” over the frontal electrode cluster at 205–329 ms (Fig. 4, left) and over central and posterior electrodes at 434–656 ms (Fig. 4, middle and right). The result confirms rapid adaptation of outcome processing to the declared behavioral relevance, rather than physical appearance, of the outcomes.

### Increased prefrontal theta frequency reflects outcome’s contextual meaning

**Figure 5:**
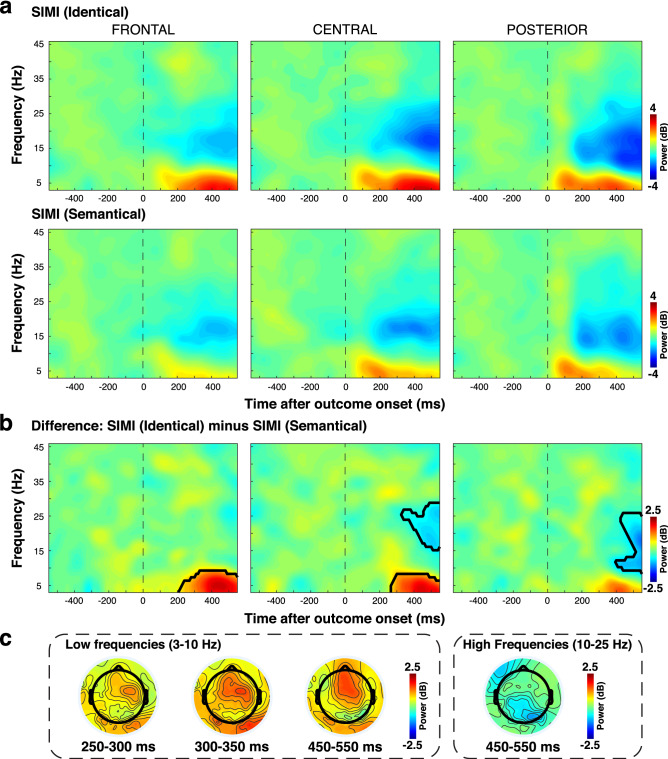
Electrodes-wise time–frequency analysis. (**a**) Power activation following outcomes SIMI presentation in condition identical (upper) and semantical (lower) at frontal (left), central (middle) and posterior (right) clusters of electrodes. (**b**) Power differences between SIMI (identical) and SIMI (semantical) at frontal (left), central (middle) and posterior (right) clusters of electrodes. Black lines indicate clusters of significant difference (p < 0.05; cluster corrected). (**c**) Topographical distribution of the power difference between SIMI (identical) and SIMI (semantical) at early and late time intervals for both low and high frequencies. The time intervals were based on the timing of distinct maps obtained in segmentation analysis.

We finally evaluated the oscillatory activity in response to SIMI stimuli in the two tasks. We used a fast Fourier transform within 300 ms Hanning tapered windows with 10 ms steps to reconstruct the time–frequency power evoked by presentation of SIMI outcomes in both task conditions. The analysis indicated early increased frontocentral low frequency (theta and low alpha, 3–10 Hz) and late decreased centroparietal high frequency (high alpha and beta, 10–25 Hz) power following presentation of the two outcomes (Fig. 5a). This finding is consistent with the midfrontal theta and beta suppression previously described following presentation of negative outcomes^41^. In our study, the evoked power was stronger when the outcomes required subsequent behavioral adaptation. Direct comparison revealed significantly stronger frontocentral power from 200 ms on and greater beta suppression around 400–550 ms after the presentation of outcomes SIMI in condition “identical” than in condition “semantical” (*p* < 0.05, cluster corrected; Fig. 5b). The topographical configuration confirmed that these differences were distinctly expressed over frontocentral (theta) and centroparietal (beta) areas (Fig. 5c).

**Figure 6:**
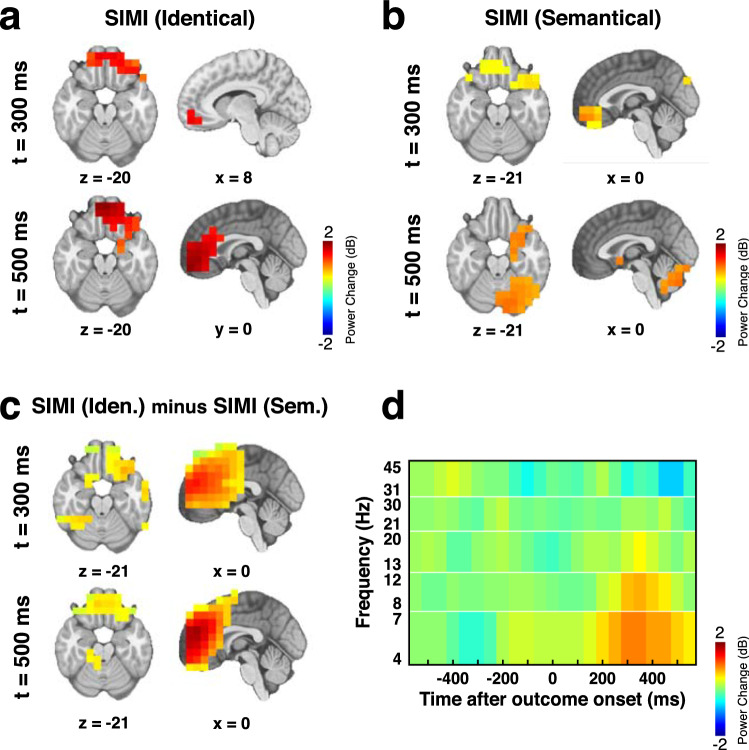
Source-space time–frequency analysis. Brain regions showing a significant (p < 0.05; cluster corrected) modulation of the power in the theta band at 300 and 500 ms following presentation of outcomes SIMI in condition (**a**) “identical”, (**b**) “semantical”; (**c**) difference between SIMI in both conditions. (**d**) Time and frequency distribution of the power difference between SIMI in both conditions observed in the right OFC. One voxel has been selected to illustrate this power distribution. The y-axis represents the frequency bands: theta (4–7 Hz), alpha (8–12 Hz), low beta (13–20 Hz), high beta (21–30 Hz) and gamma (31–45 Hz).

To evaluate the spatial distribution of the power across the time and frequencies, we ran an independent spatial beamformer analysis^42^. The spatial beamformer used the properties of the recorded signal to determine its localization within a brain model (see “Methods”). The analysis indicates significantly increased theta power in the OFC at 300 ms in response to SIMI outcomes in both “identical” (Fig. 6a) and “semantical” (Fig. 6b) conditions. At the later stage, from 500 ms on, theta activity was extended to the medial prefrontal cortex (mPFC), lateral prefrontal cortex (LPFC) and medial temporal lobe (MTL) following presentation of SIMI outcomes in the condition “identical” (Fig. 6a). During the same period, outcomes SIMI in the condition “semantical” induced significantly increased theta power in the MTL and the cerebellum (Fig. 6b). A direct comparison confirmed that outcomes SIMI in condition “identical” elicited significantly more theta and alpha activity than in condition “semantical” over prefrontal regions, including the OFC, ACC, medial prefrontal cortex (mPFC) and lateral prefrontal cortex (LPFC) (Fig. 6c). This difference was observed from 250 ms after the outcome onset for the theta band, and from 300 ms for the alpha band (Fig. 6d).

## Discussion

This study reveals a fascinating combination of precision and flexibility in human outcome processing. Stimuli closely resembling, but not identical with, a target stimulus representing the anticipated outcome, very rapidly, at about 200 ms, induced a frontal potential otherwise evoked by unequivocally different stimuli. From about 200 to 260 ms on, these stimuli were treated according to their learned behavioral relevance. Specifically, if similar stimuli had been declared by task instructions to be acceptable targets, they evoked a similar potential as true targets. If, however, task instructions designated them as non-targets, they were from the beginning processed like different stimuli despite their resemblance to the target stimuli. They now evoked a frontal positivity, similar to different stimuli. This positivity has previously been observed in deterministic reversal learning tasks when negative outcomes indicated a need to subsequently adapt behavior^25,27^.

Processing after this initial frontal potential was dominated by amplitude variations mainly over central electrodes, described before in response to unexpected outcomes. Stimuli that—according to task instructions—required a subsequent behavioral switch induced a central negativity culminating at about 350 ms, followed by a positivity at about 400–600 ms. The former potential (around 350 ms) may correspond to a feedback related negativity (FRN), a potential previously observed in response to unexpected outcomes^29–33^. The later potential (400–600 ms) may correspond to a P300 potential, which has been suggested to predict behavioral adaptation^31,34^ and to reflect context updating^31,35,36^. While many of these tasks involved decision making and reward processing, our study shows that these rapid processing stages also emerge in effortless situations like a deterministic reversal learning task devoid of any tangible reward value. Ambiguity provoked by stimulus similarity does not alter or retard this processing.

The present study is relevant for the understanding of the process that allows the brain to maintain synchrony of thought and behavior with current reality within a free flow of thoughts, a process called orbitofrontal reality filtering^18,37^. People failing in this capacity act according to ideas emanating from their past, but which do not relate to current reality (they typically enact previous habits), they are disoriented, and they confabulate about their recent doings and future plans^17^. Previous clinical studies showed that such patients fail to integrate the absence of anticipated outcomes into their behavior, as suggested by behavioral observation and validated by a failure to abandon previously valid, but now invalid stimuli in deterministic reversal learning^16,19^. Disorientation very strongly correlates with errors following such extinction trials^19^. The idea is that the brain constantly anticipates outcomes, possibly multiple ones at a time, and verifies their occurrence at the anticipated time^18,37^. If the anticipated event occurs, thinking about it relates to true reality; if the anticipated event does not occur, the thought does not relate to current reality. If the hypothesis is correct that the brain uses such a binary signal (anticipated event occurs, yes/no), to synchronize behavior with current reality, the precision and flexibility of deterministic outcome processing observed in the current study is indeed necessary for adaptive behavior. Imagine entering a room in the expectation of a specific person. If this precise person is not present, thoughts about actions related to this person have no current behavioral value and behavior has to be adapted. Conversely, if one expects to find some people in a room rather than a specific person, thoughts relating to this situation, irrespective of the precise people present, are valid and relate to the “now”. It appears vital that the brain is flexible in the generation of anticipations but precise in the verification of their occurrence.

Orbitofrontal reality filtering, and presumably the processing of stimuli demanding a behavioral switch in present study, depends on intact posterior medial orbitofrontal cortex, area 13, as derived from clinical lesions studies with patients^14,15,18^ and imaging and electrophysiological studies with healthy subjects^21,27^. The present study indicates that stimuli demanding a behavioral switch induce stronger activity in ventromedial (anterior cingulate and orbitofrontal cortex) and partly medial temporal regions, specifically in the theta frequency range, stretching over a prolonged period between about 200 and 600 ms. Indeed, medial frontal theta has previously been suggested to reflect a response to unexpected negative outcomes^41,43^ but also seems to be the preferred frequency of communication between the orbitofrontal area and the medial temporal area^44–46^. Indeed, an imaging study using H_2_-^15^O-PET showed particularly strong orbitofrontal activity in a deterministic reversal learning task when feedback was relevant for subsequent behavior, but of the medial temporal lope when the outcome of trials had to be stored for subsequent choice^14^, underscoring the importance of the frontal-medial temporal interaction.

In conclusion, this study indicates flexible adaptation of anticipations and precise verification of outcomes by the human brain, presumably guiding behavior through orbitofrontal-medial temporal interaction. These qualities are compatible with a generic outcome monitoring system subserving orbitofrontal reality filtering.

## Materials and methods

### Participants

Twenty-four healthy subjects with no history of neurological or psychiatric illness participated in this study. Two participants were excluded due to task incomprehension or antidepressant consumption (20 mg/day of fluoxetine). The twenty-two remaining subjects (13 females, aged 23.5 ± 3.1 years) were right-handed according to the Edinburgh Handedness Inventory^47^. Each participant gave written informed consent and was paid 20 Swiss francs per hour to participate. This study was approved by the regional ethics committee of the Canton of Geneva and performed according to the Declaration of Helsinki.

### Task

Participants performed a modified deterministic reversal learning task (Fig. 1a). Trials started with two colored squares (red/green) randomly positioned on the left or right side of the screen. Subjects were instructed to predict behind which one of two colored squares a target object was hidden by pressing a button: index finger of the right hand for the left square; middle finger of the right hand for the right square. As soon as they had pushed the button, a fixation cross appeared in the middle on the chosen square. After 1000 ms, the outcome—a meaningful design—was presented and remained for 800 ms, after which the screen turned black. After 500 ms the next trial started. Stimuli were presented at a distance of 0.8 m; the two rectangles covered a visual angle of about 18°.

There were three possible outcomes, presented as line drawings of objects^26,48^ (Fig. 1b): (1) the expected primary target object (e.g., a hat), called “SAME”; (2) an object having no resemblance with the target objects, called “DIFF”; (3) an object from the same semantical group and strongly resembling the primary target object in terms of overall form and visual complexity, called “SIMI”.

There were two conditions which differed with respect to the instructions (Fig. 1c). In both conditions, participants had to determine behind which one of two colored squares a target object was hidden. They were instructed to base their choice on the last outcome and refrain from guessing. The two conditions were: (1) In the first condition, named “identical”, participants were instructed to indicate the square hiding a distinct object, e.g. a specific hat (“indicate where THIS hat is hidden”). They should then continue to choose the same square until the precise object failed to occur, as indicated by the appearance of another object. If this happened, they should switch to the alternate square on the next trial. In this task condition, SIMI stimuli (e.g., another hat) would indicate absence of the target object and motivate a switch. (2) In the second condition, named “semantical”, participants were asked to indicate the square hiding a specific type of object (“indicate where A hat is hidden”). In this case, a SIMI stimulus would indicate a correct choice and motivate continued choice of the same square. In both conditions, SIMI differed from the principal target outcome (SAME) by the fact that the latter were six times more frequent. The task had six blocks (three in condition identical; three in condition semantical) with 240 trials each, of which 30 were the SIMI stimuli, 30 were DIFF stimuli, and 180 were SAME stimuli. Each block used a different triplet of objects (SAME, SIMI, DIFF; Fig. 1c), randomly chosen among six sets of different categories (Crowns—Lettuce; Drums—Pumpkin; Flowers—Tie; Hats—Cake; Mushrooms—Stool; Owls—Barrel). Each triplet of objects was presented at the beginning of each block with the corresponding condition-specific instructions. SIMI or DIFF stimuli occurred after every 2–6 consecutive SAME trials. The blocks’ presentation was counterbalanced across the subjects to avoid order effect. The experimental paradigm was programmed under Matlab (The MathWorks Inc.) using the Psychophysics Toolbox extensions^49–51^.

### Behavioral analysis

Performance was evaluated as the proportion of trials in which the subjects selected the correct square according to task condition. In addition, two types of errors were evaluated. Extinction errors were the proportion of trials in which subjects continued to choose the incorrect square after a reversal. Association errors were the proportion of trials in which the subjects incorrectly switched to the alternate square after a correct outcome. Accuracy and reaction times were compared with a 2 × 3 repeated measure ANOVA (rmANOVA) using conditions (semantical or identical) and types of trials (SAME, SIMI, DIFF) as factors. Post hoc paired t-tests were Bonferroni corrected and the effect sizes were reported as the partial eta-squared (*η*^2^). In the case of sphericity violation, we applied a Greenhouser–Geisser correction. Statistical analysis was performed using IBM SPSS Statistics for Windows, version 25.0 (IBM Corp.).

### EEG acquisition and preprocessing

Electrophysiological brain activity was recorded using a 156-channel BrainVision actiCHamp system (Brain Products CmbH, Germany) sampled at 1000 Hz and impedance at each electrode was kept under 20 kΩ. Electrode locations were recorded for each subject using Captrack camera and software (Brain Products CmbH, Germany). Prior to artifact checking, the EEG signal was bandpass filtered (1–45 Hz) using a linear finite impulse response (FIR) filtering and re-referenced to the average reference. We parcelled the signal into epochs from − 700 to + 700 ms after the outcome onset. In addition to a ± 100 µV automated rejection criterion, epochs were visually checked for artifacts, such as eye blinks, muscular contractions, and other artifacts. Bad channels were interpolated from neighboring ones using a spline interpolation. Artifact-free epochs were averaged for each subject and for each type of trial to compute event-related potentials (ERPs). We did not apply a baseline correction in the ERP analysis because our paradigm may contain expectation-related low-frequency activity that could influence post-stimulus ERP topography when the data is baseline-corrected^52–54^. However, we did apply baseline correction in time–frequency analysis in order to normalize event-related changes in the power activity relative to the baseline, which is standard in the calculation of event-related spectral perturbations^55^.

The number of epochs retained for analysis was similar for all trial types (ANOVA, p > 0.05): 55.91 ± 11.36 (mean ± sd) epochs for SAME, 50 ± 10.91 for DIFF and 53.73 ± 12.31 for SIMI in the condition “semantical”; 53.91 ± 12.98 for SAME, 51.36 ± 11.98 for DIFF and 51.32 ± 13.19 for SIMI in the condition “identical”. Preprocessing steps were performed under Matlab, using a combination of EEGlab toolbox^56^ Cartool software (cartoolcommunity.unige.ch) and personal scripts.

### Waveform analysis

We first performed a global waveform analysis covering all electrodes. Amplitude differences of ERPs between the different types of trials for both conditions were compared with a 2 × 3 rmANOVA using conditions (semantical/identical) and trial types (SAME/SIMI/DIFF) as factors. We retained as significant amplitude differences at *p* < 0.01 for ≥ 20 ms over at least five contiguous electrodes^57,58^.

Similar to earlier studies^25,27,28,39^, we next performed a focused waveform analysis over three clusters of electrodes (rather than single electrodes), each averaging five electrodes, over frontal (AFp1, AFp2, AFz, AFF1h, AFF2h), central (Cz, CCP1h, CCP2h, FCC1h, FCC2h), and posterior (POz, PPO1h, PPO2h, POO1, POO2) regions. Electrode positions corresponded to the 160Ch Standard Electrode Layout for actiCHamp based on the 10/20 system. The averaged ERPs were compared using paired t-test. To account for multiple testing, we employed a cluster correction^59^, in which all contiguous time frames (1 ms) that exceeded a significance level of 5% were grouped into one cluster. We kept only clusters with a size greater than 95% of the clusters’ distribution obtained with the 5000 randomized permutations.

The rmANOVA was performed with the Statistical Toolbox for Electrical Neuroimaging (STEN) developed by Jean-François Knebel and Michael Notter (http://doi.org/10.5281/zenodo.1164038), and the cluster waveform analysis with a custom script in Matlab software.

### Electrode-wise time–frequency analysis

Spectral decomposition of the signal over time was determined with a time–frequency analysis based on a short-time Fourier transform. Fourier coefficients were estimated for frequencies between 3 and 45 Hz with 1 Hz step, using a 300 ms fixed hanning tapered time windows slided every 10 ms, reducing the 1400 ms long epochs at − 550 to + 550 ms. The time–frequency power values were log-transformed and baseline corrected by subtracting a pre-stimulus log-transformed baseline power average from − 550 to 0 ms. Then, the obtained power (dB) was averaged within three clusters of frontal, central and posterior electrodes, similar to the waveform analysis. Power was estimated for each epoch in every subject and then averaged for statistical analysis. We then compared the power induced by SIMI trials in condition “identical” and SIMI trials in condition “semantical”. To further correct for multiple testing, we employed similar cluster correction as described in cluster waveform analysis section. For illustrative purposes, we evaluated the power at each electrode to create the associated topographical power distribution. Analyses were performed using Matlab software in combination with personal scripts.

### Source-space time–frequency analysis

Neuronal oscillatory modulations induced by SIMI trials in condition “identical” and in condition “semantical” were assessed within the source space. Event-related power changes were calculated using 1400 ms epochs projected on a lead potential with 10 mm voxels using a scalar minimum variance beamformer^42^. The lead potential was computed using a spherical head model with anatomical constraints^60^ based on the standard Montreal Neurological Institute (MNI) brain warped according to the digitized electrodes’ location. Signals were Fourier-transformed using a sliding Hanning window of 300 ms fixed-width shifted in time steps of 50 ms and projected to source space as previously described^61^, reducing the initial epochs length at -550 to + 550 ms. At each voxel, power was computed in each time window across all trials for the following frequency bands: theta (4–7 Hz), alpha (8–12 Hz), low beta (13–20 Hz), high beta (21–30 Hz), and gamma (31–45 Hz). Time–frequency power values were log-transformed and baseline corrected by subtracting a pre-stimulus log-transformed baseline power average from − 550 to 0 ms. Power (dB) maps were spatially normalized to the canonical Montreal Neurological Institute (MNI) template. Voxel-level powers were first compared within both types of trials against the null hypotheses of zero change and then compared between subjects using statistical non-parametric mapping (SnPM). Multiple testing was corrected with a cluster correction based on 5000 randomized permutations such that clusters were spatially larger than 95% of the clusters obtained during permutation. Analyses were performed using the software Matlab with the open-source toolbox NUTMEG^62^.

## Data Availability

Participants consent did not include public data sharing. Study data are available from the corresponding author upon reasonable request and on the condition of authorization by the Ethical Committee.

## Code availability

Task code is available at https://doi.org/10.26037/yareta:ojb6hz2ilzf4vaan25dfxwhyfi. Preprocessing was performed using a combination of EEGlab toolbox^56^ Cartool software (cartoolcommunity.unige.ch) and personal scripts. Waveform analysis was performed using STEN software (http://doi.org/10.5281/zenodo.1164038) and Beamforming analysis with NUTMEG^62^. Matlab code for other analyses can be obtained from the corresponding author upon reasonable request.
